# Primary Health Care in transitional care of people with stroke

**DOI:** 10.1590/0034-7167-2024-0468

**Published:** 2024-07-29

**Authors:** Adriana Bitencourt Magagnin, Kenia Lara da Silva, Giane Zupellari dos Santos Melo, Ivonete Teresinha Schulter Buss Heidemann

**Affiliations:** I Universidade Federal de Santa Catarina. Florianópolis, Santa Catarina, Brazil; II Universidade Federal de Minas Gerais. Belo Horizonte, Minas Gerais, Brazil; III Universidade do Estado do Amazonas. Manaus, Amazonas, Brazil

**Keywords:** Stroke, Primary Health Care, Transitional Care, Continuity of Patient Care, Comprehensive Health Care, Accidente Cerebrovascular, Atención Primaria de Salud, Integralidad en Salud, Cuidado de Transición, Continuidad de la Atención al Paciente

## Abstract

**Objectives:**

to understand the role of Primary Health Care teams in caring for people with stroke after hospital discharge.

**Methods:**

single case study, with integrated units of analysis, with a qualitative approach. Data triangulation occurred through interviews with professionals and family caregivers involved in transition of care, in addition to direct observations in rounds and document analysis. For the analyses, the analytical strategies of theoretical propositions and construction of explanations were used, with the help of ATLAS.ti^®^.

**Results:**

the importance of counter-referral, the role of community health workers and the multidisciplinary team, health promotion, secondary prevention, home visits as a visceral attribute and nurses as care managers are evident.

**Final Considerations:**

the high demand on teams and the Social Determinants of Health interfere with adequate continuity of care. Transitional care programs that enable continuity of care are recommended.

## RESUMEN

**Objetivos:**

comprender el papel de los equipos de Atención Primaria de Salud en la atención a personas con accidente cerebrovascular después del alta hospitalaria.

**Métodos:**

estudio de caso único, con unidades de análisis integradas, con enfoque cualitativo. La triangulación de datos ocurrió a través de entrevistas con profesionales y cuidadores familiares involucrados en la transición del cuidado, además de observaciones directas en rondas y análisis de documentos. Para los análisis se utilizaron las estrategias analíticas de proposiciones teóricas y construcción de explicaciones, con ayuda de ATLAS.ti^®^.

**Resultados:**

se evidencia la importancia de la contrarreferencia, el papel del Agente Comunitario de Salud y el equipo multidisciplinario, la promoción de la salud, la prevención secundaria, las visitas domiciliarias como atributo visceral y el enfermero como gestor del cuidado.

**Consideraciones Finales:**

la alta exigencia de los equipos y los Determinantes Sociales de la Salud interfieren con una adecuada continuidad de la atención. Se recomiendan programas de atención de transición que permitan la continuidad de la atención.

## INTRODUCTION

Stroke remains the second leading cause of death and the third leading combined cause of death and disability in the world^(1)^. Through health promotion and management of risk factors, the disease can be preventable in around 80% of cases, highlighting the importance of Primary Health Care (PHC). After the event, the performance of this service is also elementary, aiming at secondary prevention, including adequate management of hypertension, diabetes mellitus, dyslipidemia, high triglyceride levels, smoking cessation and anticoagulation in cases of arrhythmias, such as atrial fibrillation^(2)^.

Even in health systems in developed countries, care after stroke presents weaknesses involving the coordination between services. After hospitalization, care is assigned to PHC teams, who assist the person over many years, affirming the necessary guarantee of access to care after the event and configuring transition of care as a challenge in health systems^(3)^.

However, there is a gap between people’s needs and the services offered, including teams’ lack of knowledge about the disease and its specificities, mainly related to invisible disabilities, which go beyond the physical aspects, such as difficulties faced with memory, problems with concentration, fatigue and other social aspects^(4)^.

Integration and communication between healthcare services that operate in the Line of Care for people with stroke is elementary, mainly because they play an important role in planning and coordinating care. PHC services usually have prior knowledge about people in their territory and can take actions that interfere with health conditions and determinants^(4)^.

However, the hospital team also has relevant information regarding the injury that occurred, making it the first place to plan care after a stroke. Although professionals consider coordination with other services to be an important element, it is challenging due to frequent changes that occur in the structuring of assistance points for people experiencing the disease^(5)^.

An adequate transition of care must involve the coordination and continuity of actions provided between the different points of the Healthcare Network. Mainly in the context of chronic non-communicable diseases, transitional care is based on a complex care plan, recognizing the availability of services in maintaining people’s health goals. These are complex moments and require many arrangements, such as an established flow in the health network, family education and communication between the professionals involved^(6)^.

The Brazilian Health System (SUS - *Sistema Único de Saúde*) is based on its Primary Care services, and Family Health Strategy is characterized as a program aligned with its guidelines, highlighting actions in health promotion, prevention and person-centered care. PHC has the potential to positively impact the world’s main health problems, such as reducing hospitalizations for PHC-sensitive diseases and tackling risk factors, with an eye on the Social Determinants of Health (SDH). However, the desired comprehensiveness of care can be fragile, as it is conditioned by several organizational elements, technical capacity and shaped by the population’s socioeconomic issues. Although PHC has its role in promoting health and preventing diseases, when a person suffers from a stroke and after hospital discharge, it maintains its ordering and coordinating role in care^(7)^.

## OBJECTIVES

To understand the role of PHC teams in caring for people with stroke after hospital discharge in a city in southern Brazil.

## METHODS

### Ethical aspects

The study was conducted in accordance with national and international ethical guidelines, approved by the Research Ethics Committee. The Informed Consent Form (IFC) was obtained from all individuals involved in the study, in writing, in two copies, one for participants and the other for researchers.

### Study design and theoretical-methodological framework

This is an explanatory, descriptive and interpretative study, using the methodological strategy of a single case study, with integrated units of analysis and a qualitative approach. The method is used in the study of contemporary phenomena, seeking their understanding through data triangulation^(8)^. The “case” studied is the Healthcare Network of a municipality in southern Brazil, involving transition of care of people with stroke. The integrated units of analysis were PHC, Stroke Unit (Stroke-U) and family caregivers of people who experienced stroke.

The COnsolidated criteria for REporting Qualitative research (COREQ) protocol was respected, which protects the quality of qualitative studies in a checklist^(9)^. For theoretical guidance, comprehensiveness^(10-11)^ and transition of care frameworks were used^(6)^.

### Study setting

The study was carried out in a municipality in northern Santa Catarina, which has a public hospital for specialized care for people affected by stroke, with a Stroke-U, for care in the rehabilitation phase of the disease, called Comprehensive Stroke-U, with 21 beds. Furthermore, the PHC network is structured into 55 basic units, which, among their activities, assist people with stroke and seek to ensure continuity of care after hospital discharge. It is understood that family caregivers are an important element for the appropriate transition and, therefore, were also part of the study.

As predicted by the method^(8)^, during data collection, a key informant listed support services involved in transition of care. Thus, in addition to integrated analysis units, four other participants were included in the research, representing Digital Healthcare services, Multidisciplinary Home Care Team and Stroke Epidemiological Record.

### Data source

For participation, healthcare professionals with at least six months of experience in the workplace were included. Family caregivers of people with stroke who were discharged from hospital more than three months ago and who identified themselves as the main caregiver were included. Meanwhile, healthcare professionals who were on vacation, on leave or away for some other reason were excluded. Caregivers who perform the role formally were excluded. One professional was excluded because she was on vacation, in addition to two caregivers, who were nominated by the teams (due to not accepting to participate in the study and moving to another city). Data collection was carried out by the main researcher.

### Data collection and organization

Data collection took place between September 2021 and February 2022. Data triangulation took place through the following sources: 12 interviews with PHC professionals; six interviews with caregivers of people with stroke; 13 interviews with professionals from the hospital unit; four interviews with support services professionals; five observations of interdisciplinary rounds in hospital settings; and analysis of 28 documents (including scientific articles from the integrative review that supported the study^(12)^, Ministry of Health documents, the municipality’s Line of Care as well as instruments for hospital discharge related to transition of care). The interviews and observations lasted approximately 60 minutes.

In a hospital setting, a moment of continuing education was used to present the research, identify professionals and invite them to the study. In PHC, the initial contact occurred by presenting the proposal to managers and, finally, inviting healthcare professionals. For family caregivers, the bridge made through community health workers (CHW) stands out so that the researcher could go to the home, explain the objective of the research and invite them to participate.

A specific instrument was used for each group interviewed and for observations, addressing issues related to transition of care, perceptions of gPHC that occur in this context, understanding of the important elements for discharge and organization of services so that transition occurs smoothly safe. The interviews carried out were recorded, and a field diary was used. Non-participant observation occurred during hospital team rounds. A case study database was developed, using ATLAS.ti^®^, to manage and organize sources of evidence.

To guarantee anonymity, participants were identified with code names. “PHC” was used for professionals in this service, “Stroke-U” for hospital team professionals, “FC” for family caregivers and “SS” for support services.

### Data analysis

The use of a software allowed identifying citations relevant to the study objective and, subsequently, enabled the creation of codes that were finally grouped into main categories. The analysis was anchored in the theoretical frameworks used.

Based on the methodological framework used^(8)^, the analytical strategies developed at this stage were based on theoretical proposition (maintaining the researcher’s focus on what guided him throughout the study) and case description development (which allows data organization from the establishment of important relationships and concepts for the study, seeking to build a data structure). As an analytical technique, we used the technique of constructing the explanation of the phenomenon and the causal links^(8)^.

## RESULTS

Collection involved 35 participants. Among the 29 professionals, only four were male. Regarding age, the age group between 41 and 50 years old predominated, with representation of ten participants, followed by eight professionals between 31 and 40 years old, ten, from 41 to 50 years old, and five, over 51 years old.

In relation to job tenure, it was clear that 16 participants had experience for more than two years in the analyzed unit of analysis and seven participants declared experience of less than one year. Regarding length of training, only three reported a period of less than two years. Specifically in PHC, as the focus of this manuscript, seven professionals had experience of less than two years in the unit investigated.

As for family caregivers, among the six participants, only one interviewee was male. Regarding age, two participants were between 20 and 40 years old, three were between 40 and 60 years old, and only one was above this age range. In relation to the degree of kinship, four were children, one was a wife and another identified herself as a granddaughter.

This qualitative research obtained a significant volume of results, which were analyzed based on 777 citations. The categories originated were later explored, covering different nuances of PHC. In this article, we presented an in-depth analysis of the category “Action of Primary Health Care in transition of care”, which was related to 298 citations, due to its highlighted relevance in the context of transition of care and to respond to the proposed objective.

PHC involvement in continuity of care for people with stroke after hospital discharge was discussed with all study participants, and the results portray some challenges in this point of care, such as the departure from the basic precepts of the service, the search for health promotion and disease prevention, due to a scenario that focuses on curative issues that require immediate care after becoming ill. The high demand in the units’ spontaneous demand generates anxiety among professionals who are often unable to provide longitudinal care, as reported in the speech below.

*And many still do not understand the current concept of Primary Health Care. They do not understand that it is prevention. We just work on the problem. And I think we’re losing this essence of Primary Health Care. Many patients describe this as an ER* [Emergency Room], *so I think we’re losing that a little.* [...] *I’m tired of putting out fires, I want to meet the family I assist.* (PHC7)

Professionals from other services also understand the necessary continuity of care in PHC as the only way to impact the reduction of stroke incidence and sufficient secondary prevention, avoiding disease recurrence.

*If you increase beds in the hospital, it means that your Primary Care is bad. However, for your Primary Care to improve, when you return a patient, what is expected is to control the risk factors, to monitor this case to prevent it from recurring, otherwise the hospital will have to start increasing bed.* (SS2)

According to the municipal Line of Care, a medical professional or nurse at the health unit must provide first care. This healthcare must promote continuity of monitoring, including appropriate treatment according to the etiology of the event, identifying and acting on risk factors that are modifiable and enabling a person’s inclusion in existing programs, such as groups for people with hypertension and diabetes, encouraging physical activity and controlling smoking. In the speeches, it was possible to observe a comprehensive view of participants, which permeates medicalization.

*I try to show patients that it goes far beyond medication* [...] *if we inform patients, it is much easier for them to follow what we are saying, right? If we ask for a test that they don’t even know what it is for, it is very unlikely that patients return.* (PHC7)

Professionals perceive transition of care as necessary continued assistance that, even after dehospitalization, remains in the network with the watchful eye of PHC. The statement below portrays what professionals list as priorities after stroke and hospital discharge.

*The main thing is to take care of comorbidities to try to avoid new events. Many of them are not aware that good control of comorbidities really means reducing the risk of events, so we really try to guide and educate a lot about the importance of controlling blood pressure, diabetes, smoking* [...]. (PHC2)

The importance of including the family in health practices and care planning is also mentioned, understanding that they have an important role in this context. Furthermore, PHC portrays the possibility of greater bonds with people and the sharing of decisions.

*We also need to prevent risk factors, because, if not, we are a strong candidate for new complications. So, for me, it is comprehensive care, it is a line of care that we must follow and that is where we find professionals at both the tertiary, secondary and primary levels. We have to bring it not vertically, but more horizontally, to be able to provide adequate quality care not only for them, but for familyiesas well.* (PHC8)

Although it is not indicated in the established flows, the multidisciplinary team’s work was brought up by participants as an important point of support in transition of care, including encouraging changes in lifestyle and rehabilitation adapted to each situation. A caregiver’s speech highlights the importance of multidisciplinary action for healthier habits.

*The mother’s glucose levels were very low. We had already tried to change our diet in other ways, but not radically. Now, with a nutritionist, she cut out everything: sugar, carbohydrates* [...] *diabetes is a silent disease, right, it’s a disease for which there is no miracle treatment. You need to change your lifestyle.* (FC4)

*Our physiotherapist from NASF* [Family Health Support Center] *makes home visits to patients, but it’s not that regular, especially because it’s just a physio and the demand is huge. He tries to show the family what they can use within their conditions. For patients’ improvement, the issue of mattress, positioning, changing position* [...]. (PHC7)

Organizational obstacles, such as a shortage of professionals, are cited by some participants. However, it was observed that teams make new arrangements in order to be able to offer the appropriate service to discharged persons, as in the speech reported below.

*We had an occupational therapist* [OT] *and two psychologists. Now, we have just one psychologist, but we have a very strong engagement with patients. It’s our responsibility. Sometimes, I don’t have an OT, but if I need it, the coordinator plays in the coordination group and the staff helps, you know? In the same way that we have a gynecologist and pediatrician, we help others too. The exchange between teams and the responsibility you have with patients, I think is really cool.* (PHC10)

In relation to the strategies carried out by PHC, health promotion is mentioned, mainly involving collective actions, such as the groups carried out. Although the COVID-19 pandemic made meetings impossible, professionals highlighted the importance of these actions to positively influence stroke risk factors.

The role of professional nurses stands out in statements of participants who work in PHC, rescuing the category’s comprehensive and attentive view in managing the care for people with stroke.

*No matter how many times the CHW, the doctor, everyone visits, the nurse is the one who draws up a plan for this patient. We are the ones who really try to see what the needs are and see the family as a whole* [...] *taking great care in prescribing special dressing materials, evaluating, monitoring, taking the discussion to the multi team, involving the entire team, being a little more ahead of this family. I see the nurse who coordinates all this care. We are a bit of a driver of this process. I realize that nursing also ends up doing as if it were a “Single Therapeutic Project” there, for that family, for that user, at that moment. Because she has already instructed the community health worker to come by monthly to see how she is doing; If you think there is a need for a technician to go, we can also provide that outcome. We end up carrying out some controls, which are part of the nursing profile itself. We have this slightly broader view of care.* (PHC8)

*Normally, the nurse ends up receiving this information first* [counter-referral], *especially because we end up working more on those scheduled. They end up receiving counter-referral and schedule a home visit so they can assess patients. Sometimes, even an appointment at the unit if patients are able to come. Afterwards, they end up passing this on to me, within the team, for us to either pay a visit or to make an appointment and monitor this patient.* (PHC6)

*Assessing patients and what their needs were, what their limitations and sequels were, find out how patients are doing and what care they need. From this care that we assess to create a care plan, prepare and organize it with the family.* (PHC5)

In addition to the important role of nursing in healthcare services, in Basic Health Units’ work, the bond with people and their families is added, allowing the team to become a reference to the community when seeking guidance or any other type of care. However, some barriers are encountered by family members when they seek help and information, as described below.

*The health center team is lacking. One of the biggest difficulties we have nowadays is when you get used to a team, they go to another place, then you have to get used to it all over again. I was already used to that nurse, she was the person who helped us the most before his stroke and after a lot of things I still talked to her, then she went to the other unit* [...] *this month, I was afraid I wouldn’t able to get the materials. From now on, I no longer know which nurse will take care of him* [family member who had the stroke]*, so I will have to start all over again. Of course, there must be a lot of information in the system. We get a little lost. Just like the doctor, if you go to them twice, by the third time you arrive, they already know you a little, right? Then you move and start all over again. It makes it a little difficult for us.* (FC5)

With the construction of a Stroke Line of Care in the city studied, we sought to improve continuity of care and comprehensive assistance for the affected person, including upon their return home. The first days after hospital discharge represent the most stressful time for families, with many doubts and insecurities arising regarding the care to be provided. Professionals who work in hospital settings highlight the necessary brevity of continuity of care provided by PHC, as observed in team meetings. During collection at one of the support services, a professional reports that, during follow-up telephone calls, she observes that some people are unable to access services for consultation within the recommended time.

*We need to think of some strategy, to have someone who is the reference for this patient, a professional who will help them on this journey, because they will arrive home full of doubts and they will not be able to call the Stroke Unit and speak to the nurse to give them the support they need. They will go to Primary Care, they will be welcomed, but they will enter a schedule that may not answer their immediate questions, and appointment will be in 15 or 20 days.* (SS2)

In addition to guaranteeing access to PHC, there is an urgent need for comprehensive care, which also meets the specificities of actions after stroke and its implications. It involves user and family engagement in care, going beyond the biomedical model and focusing only on medicalization and requesting tests, which may require an effort from the professional who assists them, to raise awareness about continuity of care, secondary prevention and others necessary healthy habits.

*And I don’t just put the blame on the population. Sometimes it is our blame. We also do it wrong. Sometimes, it’s much easier for you to hand them a sheet with a complete blood count than having to explain to them that they need to eat better.* (Stroke-U7)

[...] *a nutritionist’s guidance at discharge for nutritional re-education is very basic guidance, very general, it is not individualized. So, often, it doesn’t have any effect on changing habits, right? There needs to be something, closer monitoring, a program, group or individual program with goals.* (Stroke-U1)

In the context of transition of care, one point mentioned by participants was regarding the role of CHWs in this transition of care process. The statements below portray this professional as an important figure in Family Health teams, as they promote bonding, have knowledge of the territory and recognize problems, providing other health unit members with relevant information that can be transmitted prior to counter-referral reporting.

*We tend to use community health workers a lot, who also monitor users. They also make visits and end up discovering* [people after a stroke]. *Sometimes, it’s not even a patient who had a stroke here, but it’s a patient who had a stroke in another city and ended up coming because of their family, and we end up finding out about this through CHW.* (PHC6)

*Generally, we end up finding out through CHW that a patient was hospitalized because of a stroke, so we ask a family member to call the unit to schedule a follow-up appointment after hospitalization.* (PHC11)

*The health center knows us a lot. They’re really nice at getting things, you know. So, we chat a lot, we talk about other places, about academias where we already know each other. They are very attentive here at home. They know that my son is autistic, that his father had a stroke, and they ask about them. I can say that they went out of their way to get physio, to get things done. She is very, very attentive. They are passing by directly, so when there is a question, I ask.* (FC3)

Participants also reported that, despite the relevance of CHW for family health, the shortage of professionals, the lack of coverage of micro areas and other tasks carried out by the category rule out the possibility of this professional’s ideal performance in the territory. The statements regarding the difficulties faced with the COVID-19 pandemic are supported among professionals and caregivers.

*We have community workers who visit homes, and, due to the pandemic, this routine has changed*. (PHC5)

*With the pandemic, a lot has changed. They weren’t on the street, we didn’t know something, and then the family member came here to explain. Even when I needed some referrals, they ended up coming* [...]. *The CHW, due to lack of staff, started to stay inside the station without being able to make visits. Here they had nothing else to bring to us, what was happening on the street. We had some things that the family member would later bring, but CHWs were not always there.* (PHC3)

*With the pandemic, there were no more visits from CHWs, and these teams are now very lacking.* (FC5)

Even though there is an established Line of Care and the creation of a counter-referral flow between hospital and PHC services, the recognition of stroke as an injury as important as other care demands is highlighted compared to children’s health, prenatal care, tuberculosis, among others.

*We don’t have this culture, in our country, of valuing Primary Care. We understand how rates improve, mortality decreases, how well you control hypertension, control diabetes well, knowing that you will resolve 80% of your patient’s reasons for consultation in Primary Care, you strengthen the role of family doctor, of nurse, of a team that works there on the strategy.* (Stroke-U7)

When questioning professionals about Line of Care, a weakness in material knowledge and reading was identified, justified by some participants due to the high demand for care and work overload.

*I couldn’t read it. There’s so much that arrives, so reading all that and another 25 or 30 patients a day gets complicated, you know?* (PHC4)

## DISCUSSION

PHC is the reference service for users after hospital discharge, as the team is located at a point in the network that provides greater bonds and approaches to people and their families, in addition to enabling participation in care and sharing of decisions. It is important for families to identify where they can seek support and who are the professionals they refer to for assistance with care after discharge, indicating which services are available in the network for the rehabilitation process. Actions also include support and guidance for demands that go beyond physical issues, often forgotten by professionals^(2,5)^.

Other elements are relevant in the perception of those who survived a stroke, such as being able to return to study, having autonomy for basic activities of daily living, maintaining socialization with friends and family and understanding about the disease. The appropriate transition of care for PHC must promote relevant information regarding health conditions and risks of a new event, guide the correct use of medications and encourage healthy habits, such as physical activity, smoking cessation and dietary care^(4)^, a fact that was observed in the case studied.

Some initiatives were listed by participants involving strategies for healthier lifestyle habits, such as guidance on nutrition, blood pressure control and smoking. A Brazilian study assessed the effects of the *Programa Academia da Saúde* (Health Academy Program) on spending on hospitalizations for stroke, and, among its findings, municipalities that joined the program reduced costs, which reinforces the importance of health promotion policies aimed at changes in lifestyle^(13)^.

Actions that are not restricted only to people at higher cardiovascular risk are supported. Non-pharmacological interventions, such as encouraging lifestyle changes and healthy public policies, are reinforced by the literature as beneficial strategies. Healthcare services can also provide opportunities for the exchange of experiences between family caregivers. This strategy can help them collectively find tools to cope throughout care and rehabilitation^(14)^, including skills development, giving families a voice in the face of the challenges left by the disease. A change of perspective is necessary for those working in healthcare, understanding that all people with any risk of stroke and cardiovascular disease should be targeted for health promotion, not just groups considered to be at higher risk^(15)^.

Aiming to increase the resolution of primary services, multidisciplinary teams expand people’s healthcare, incorporating comprehensive care into its essence. Furthermore, CHWs are described in this study as a bridge between the community and the services provided by health facilities. Known as a category that works to prevent diseases and promote health, it also suffered interference with the update of the Brazilian National Primary Care Policy. The mischaracterization of the role of this professional occurs with the arrival of procedures within their scope of action, such as measuring vital signs and simple dressing techniques^(16)^, which may weaken other actions in the territory.

As part of the scope of Basic Health Units, home visit programs are a unique practice of Primary Care, especially when it involves the multidisciplinary team, which assists in the adequate transition of care due to its possibility of broadening the view of comprehensiveness of care to aspects that go beyond the disease^(17-18)^.

A randomized clinical trial^(19)^ found that the group of people and family members who received visits from nursing professionals at home after discharge had greater access to the hospital outpatient service, which can be justified by the greater link with the network point and effectiveness in guidelines. The visits included health education, guidance on the system and real demonstrations about care for skills development. Furthermore, the family was informed about services where they could obtain assistance with materials and supplies for care, which was the families’ main concern in the first days after discharge.

During a home visit, it is also possible to identify the SDH, including assessment of adequate nutrition, socioeconomic situation, physical structure and family support^(20)^. Furthermore, providing guidance adapted to family realities strengthens engagement in care and sharing decisions^(21)^. Although visits are part of a teams’ scope, during collection, it was not observed how the teams plan to continue monitoring.

Regarding health information, it is recommended that communication be clear, including which services are available to people, understanding the context in which they live. Health technology services are well assessed, such as the counter-referral used between hospital and PHC services, as they can enable routine monitoring of new stroke cases and organization of teams to care for these people. Furthermore, telehealth can be advantageous in collaboration between professionals^(22)^.

The persistence of mortality rates in some regions of the world suggests more effective public policies, mainly focusing on primary and secondary prevention. On the other hand, it is understood that countries with a reduction in crude mortality rates over the years have developed stroke management and treatment strategies, such as the creation of stroke units and identification of the mildest cases^(23)^. This reinforces hospital teams’ work, as observed in the study, which focuses on discharge and caregiver preparation from the moment of hospitalization.

Well-established Lines of Care and service flowcharts are essential to ensure adequate transition, although they are not sufficient. Care planning must be carried out at all points that provide assistance to individuals with a stroke, and the quality provided in the acute phase of the event must be continued at other points in the Healthcare Network.

Although understanding regarding the role of PHC services was important, professionals are faced with difficulties in their routines. This factor also compromises the experience and access of people and family members living with stroke. The obstacles identified support the literature, such as the high demand at the doors of the units for specific care, overcrowding, difficulties in accessing rehabilitation, geographic limits, structural issues and team sizing, the impact of the pandemic and change in health policies. It is a context that challenges the multidisciplinary team coordination for adequate continuity of care in the complexity of stroke, especially as it requires different approaches^(3)^.

Health is determined and conditioned by several factors that go beyond behavioral changes. The paths to advances in health are based on health promotion, preventive measures and better living conditions. It requires a positive outlook on health, healthy public policies, reorganization of the health network, community involvement in decision-making, healthy environments and development of personal skills^(24-25)^.

### Study limitations

The study was carried out in a city recognized for its care for people with stroke, qualified under Ordinance 665 of 2012 as a Type III Emergency Care Center^(26)^. Furthermore, it has a multidisciplinary residency program and constantly encourages the improvement of care for people with stroke, which strongly contributes to transition of care. The limitation of this study includes the generalization of data to other realities. Furthermore, the implications of the COVID-19 pandemic on teams’ routines were observed, interfering with professional activities and, in short, determining findings that could be different in other health conditions.

### Contributions to nursing, health and public policy

The research results expand nursing action perspectives in promoting health in a hospital setting, valuing the role of transition nurses. It is relevant to professional training and multidisciplinary work in the SUS, with a view to the Stroke Line of Care and care in the Healthcare Network. The research provides reinforcement of understanding of health management regarding the assessment of results of established flows, instigating coordination between Healthcare Network points, with the creation of public policies to reduce stroke mortality and coordinate care after discharge from PHC.

The first few days after discharge are the most delicate time for families, requiring timely action to identify the family’s needs. It reflects on the possibility of transitional care programs that enable continuity of care without there being gaps between points in the network. In addition to creating Lines of Care, continuing education is also necessary to train teams for assistance, enabling transition of care.

## FINAL CONSIDERATIONS

PHC’s role in caring for people after stroke includes opportunities for access to services, secondary prevention and health-promoting strategies aimed at empowering and empowering people, improving their lifestyles, surveillance carried out by CHWs and multidisciplinary action to guarantee comprehensive care. Furthermore, the leading role of families and professional nurses as care managers is emphasized.

Challenges include high workload in units, SDH and the COVID-19 pandemic. New studies are recommended in other realities, including the identification of different arrangements in the Line of Care for people with stroke that can identify relevant strategies to guarantee transition of care.
