# ERRATUM: Primary Health Care in transitional care of people with stroke

**DOI:** 10.1590/0034-7167.202679e0420230468

**Published:** 2026-05-27

In the article “Primary Health Care in transitional care of people with stroke”, with DOI number: https://doi.org/10.1590/0034-7167-2024-0468, published in Revista Brasileira de Enfermagem, 2024;77(3):e20230468:

Where it read:

https://doi.org/10.1590/0034-7167-2024-0468

It reads:

https://doi.org/10.1590/0034-7167-2023-0468

Prof. PhD Dulce Barbosa

*Editor in Chief*

