# Supplementary material for: Primary Health Care in transitional care of people with stroke
Source: Rev Bras Enferm. 2024 Jul 29;77(3):e20230468. doi: 10.1590/0034-7167-2024-0468 (PMC11290722; doi:10.1590/0034-7167-2024-0468)
Supplement: Translation

# Translation: Atenção Primária à Saúde na transição do cuidado de pessoas com Acidente Vascular Cerebral

**DOI:** 10.1590/0034-7167-2024-0468pt

**Authors:** Adriana Bitencourt Magagnin, Kenia Lara da Silva, Giane Zupellari dos Santos Melo, Ivonete Teresinha Schulter Buss Heidemann

**Affiliations:** I Universidade Federal de Santa Catarina. Florianópolis, Santa Catarina, Brasil; II Universidade Federal de Minas Gerais. Belo Horizonte, Minas Gerais, Brasil; III Universidade do Estado do Amazonas. Manaus, Amazonas, Brasil

**Keywords:** Acidente Vascular Cerebral, Atenção Primária à, Saúde, Cuidado Transicional, Continuidade da Assistência ao Paciente, Integralidade em Saúde

## Abstract

**Objetivos:**

compreender a atuação das equipes de Atenção Primária à Saúde no cuidado às pessoas com Acidente Vascular Cerebral após a alta hospitalar.

**Métodos:**

estudo de caso único, com unidades de análise integradas, de abordagem qualitativa. A triangulação dos dados ocorreu através de entrevistas com os profissionais e cuidadores familiares envolvidos na transição do cuidado, além de observações diretas em *rounds* e análise documental. Para as análises, foram utilizadas as estratégias analíticas das proposições teóricas e construção de explicações, com auxílio do *software ATLAS.ti* ^®^.

**Resultados:**

evidencia-se a importância da contrarreferência, o papel do Agente Comunitário de Saúde e da equipe multiprofissional, promoção da saúde, prevenção secundária, visita domiciliar como atributo visceral e enfermeiro como gestor do cuidado.

**Considerações Finais:**

a alta demanda das equipes e os Determinantes Sociais da Saúde interferem na continuidade adequada do cuidado. Recomendam-se programas de cuidados de transição que possibilitem a continuidade da assistência.

## INTRODUÇÃO

O Acidente Vascular Cerebral (AVC) continua sendo a segunda principal causa de morte e a terceira principal causa combinada de morte e incapacidade no mundo^(1)^. Através da promoção da saúde e do manejo dos fatores de risco, a doença pode ser evitável em cerca de 80% dos casos, resgatando a importância da Atenção Primária à Saúde (APS). Após o evento, a atuação desse serviço também é elementar, visando à prevenção secundária, incluindo manejo adequado da hipertensão arterial sistêmica, diabetes mellitus, dislipidemia, níveis elevados de triglicerídeos, cessação do tabaco e anticoagulação em casos de arritmias, como a fibrilação atrial^(2)^.

Mesmo em sistemas de saúde de países desenvolvidos, o cuidado após o AVC apresenta fragilidades envolvendo a articulação entre os serviços. Após a hospitalização, o cuidado é atribuído às equipes de APS, que assistem a pessoa ao longo de muitos anos, afirmando a necessária garantia de acesso aos cuidados após o evento e configurando a transição do cuidado como um desafio nos sistemas de saúde^(3)^.

Todavia, há uma lacuna entre as necessidades das pessoas e os serviços ofertados, incluindo o desconhecimento das equipes a respeito da doença e suas especificidades, principalmente relacionada às deficiências invisíveis, que ultrapassam os aspectos físicos, como as dificuldades enfrentadas com a memória, problemas de concentração, fadiga e outros aspectos sociais^(4)^.

A integração e comunicação entre os serviços de saúde que atuam na Linha de Cuidado à pessoa com AVC é elementar, principalmente porque esses possuem importante papel no planejamento e na coordenação do cuidado. Serviços de APS usualmente têm conhecimento prévio a respeito das pessoas do seu território e podem levantar ações que interfiram nos condicionantes e determinantes de saúde^(4)^.

Não obstante, a equipe hospitalar também possui informações relevantes a respeito do agravo ocorrido, caracterizando-se como o primeiro local a planejar o cuidado após o AVC. Embora os profissionais considerem a coordenação com outros serviços como um elemento importante, mostra-se desafiadora, devido às frequentes mudanças que ocorrem na estruturação dos pontos de assistência às pessoas que vivenciam a doença^(5)^.

A transição do cuidado adequada deve envolver a coordenação e a continuidade de ações prestadas entre os diferentes pontos da Rede de Atenção à Saúde. Principalmente no contexto das doenças crônicas não transmissíveis, cuidados de transição fundamentam-se em um complexo plano de cuidados, reconhecendo a disponibilidade dos serviços na manutenção de metas em saúde das pessoas. São momentos complexos e requerem muitos arranjos, como um fluxo estabelecido na rede de saúde, educação para a família e comunicação entre os profissionais envolvidos^(6)^.

O Sistema Único de Saúde (SUS) é alicerçado por seus serviços de Atenção Básica, e a Estratégia Saúde da Família caracteriza-se como um programa alinhado com suas diretrizes, realçando a atuação em promoção da saúde, prevenção e cuidado centrado na pessoa. Cuidados primários em saúde têm potencial em impactar positivamente os principais problemas de saúde do globo, como a redução das hospitalizações por doenças sensíveis à APS e o enfrentamento dos fatores de risco, com o olhar aos Determinantes Sociais da Saúde (DSS). Todavia, a almejada integralidade do cuidado pode ser frágil, pois é condicionada por diversos elementos organizacionais, de capacidade técnica e moldada às questões socioeconômicas da população. Embora a APS tenha seu caráter de atuação na promoção da saúde e prevenção das doenças, diante do acometimento da pessoa com AVC e após a alta hospitalar, mantém seu papel ordenador e coordenador do cuidado^(7)^.

## OBJETIVOS

Compreender a atuação das equipes de APS no cuidado às pessoas com AVC após a alta hospitalar em um município do sul do Brasil.

## MÉTODOS

### Aspectos éticos

Estudo foi conduzido de acordo com as diretrizes de ética nacionais e internacionais, aprovado por Comitê de Ética em Pesquisa. O Termo de Consentimento Livre e Esclarecido (TCLE) foi obtido de todos os indivíduos envolvidos no estudo, por meio escrito, em duas vias, para posse do participante e das pesquisadoras.

### Tipo de estudo e referencial teórico-metodológico

Trata-se de estudo explanatório, descritivo e interpretativo, com a utilização da estratégia metodológica de estudo de caso único, com unidades de análise integradas e de abordagem qualitativa. O método é utilizado no estudo de fenômenos contemporâneos, buscando sua compreensão a partir da triangulação de dados^(8)^. O “caso” estudado é a Rede de Atenção à Saúde de um município da região Sul do Brasil, envolvendo a transição do cuidado de pessoas com AVC. As unidades integradas de análise foram APS, Unidade de Acidente Vascular Cerebral (U-AVC) e cuidadores familiares de pessoas que vivenciaram AVC.

Respeitou-se o protocolo *COnsolidated criteria for REporting Qualitative research* (COREQ), que resguarda a qualidade dos estudos qualitativos em *checklist* ^(9)^. Para direcionamento teórico, utilizaram-se os referenciais da integralidade^(10-11)^ e a transição do cuidado^(6)^.

### Cenário do estudo

O estudo foi realizado em município no norte de Santa Catarina, que conta com um hospital público para atendimento especializado às pessoas acometidas pelo AVC, possuindo uma U-AVC, para atendimento na fase de reabilitação da doença, denominada U-AVC Integral, com 21 leitos. Além disso, a rede de APS está estruturada em 55 unidades básicas, que, entre suas atividades, assistem as pessoas com AVC e buscam garantir a continuidade do cuidado após a alta hospitalar. Compreende-se que os cuidadores familiares são elemento importante para a transição adequada e, desse modo, também fizeram parte do estudo.

Conforme previsto pelo método^(8)^, durante a coleta dos dados, um informante-chave elencou serviços de apoio envolvidos na transição do cuidado. Desse modo, além das unidades integradas de análise, outros quatro participantes foram incluídos na pesquisa, representando os serviços de Saúde Digital, Equipe Multiprofissional de Atenção Domiciliar e Registro Epidemiológico de AVC.

### Fonte de dados

Para a participação, foram incluídos os profissionais dos serviços de saúde com no mínimo seis meses de atuação no local de trabalho. Foram incluídos os familiares cuidadores de pessoas com AVC que vivenciaram a alta hospitalar há mais de três meses e que se identificaram como cuidador principal. Enquanto isso, foram excluídos os profissionais de saúde que estivessem em período de férias, licença ou afastados por algum outro motivo. Os cuidadores que exercem a função de modo formal foram excluídos. Uma profissional foi excluída por estar em período de férias, além de dois cuidadores, que foram indicados pelas equipes (por não aceite na participação no estudo e mudança de cidade). A coleta dos dados foi realizada pela pesquisadora principal.

### Coleta e organização dos dados

A coleta ocorreu entre setembro de 2021 e fevereiro de 2022. A triangulação de dados se deu pelas seguintes fontes: 12 entrevistas com profissionais da APS; seis entrevistas com cuidadores de pessoas com AVC; 13 entrevistas com profissionais da unidade hospitalar; quatro entrevistas com profissionais dos serviços de apoio; cinco observações dos *rounds* interdisciplinares no ambiente hospitalar; e análise de 28 documentos (incluindo artigos científicos da revisão integrativa que sustentou o estudo^(12)^, documentos do Ministério da Saúde, Linha de Cuidado do município, além de instrumentos para a alta hospitalar relacionados à transição de cuidados). As entrevistas e observações tiveram duração aproximada de 60 minutos.

No ambiente hospitalar, utilizou-se um momento de educação continuada para apresentar a pesquisa, identificar os profissionais e convidá-los ao estudo. Na APS, o contato inicial ocorreu mediante apresentação da proposta aos gestores e, por fim, o convite aos profissionais de saúde. Aos cuidadores familiares, ressalta-se a ponte realizada através do Agente Comunitário de Saúde (ACS), para que a pesquisadora pudesse se dirigir até o domicílio, explicar o objetivo da pesquisa e convidá-los para participação.

Utilizou-se um instrumento específico para cada grupo entrevistado e para as observações, abordando questões relacionadas à transição do cuidado, percepções das lacunas que ocorrem nesse contexto, compreensão acerca dos elementos importantes para a alta e organização dos serviços para que a transição ocorra de forma segura. As entrevistas realizadas foram gravadas, e usou-se um diário de campo. As observações ocorreram de forma não participante, durante os *rounds* da equipe hospitalar. Um banco de dados do estudo de caso foi desenvolvido, usando o *software ATLAS.ti* ^®^, para gerenciar e organizar as fontes de evidência.

Para garantir o anonimato, os participantes foram identificados com codinomes. Utilizou-se “APS” para profissionais deste serviço, “U-AVC” para profissionais da equipe hospitalar, “CF” para cuidadores familiares e “SA” para serviços de apoio.

### Análise dos dados

O uso do *software* permitiu identificar as citações pertinentes ao objetivo do estudo e, posteriormente, possibilitou a criação dos códigos que, por fim, foram agrupados em categorias principais. A análise foi ancorada nos referenciais teóricos utilizados.

A partir do referencial metodológico utilizado^(8)^, as estratégias analíticas desenvolvidas nessa etapa foram a partir da proposição teórica (manter o foco do pesquisador ao que o orientou durante todo o estudo) e do desenvolvimento da descrição do caso (que permite a organização dos dados a partir do estabelecimento de relações e conceitos importantes para o estudo, buscando a construção de uma estrutura dos dados). Como técnica analítica, utilizaram-se a técnica de construção da explicação do fenômeno e os elos causais^(8)^.

## RESULTADOS

A coleta envolveu 35 participantes. Entre os 29 profissionais, apenas quatro eram do sexo masculino. A respeito da idade, predominou a faixa etária entre 41 e 50 anos, com representatividade de dez participantes, seguida de oito profissionais entre 31 e 40 anos, dez, de 41 a 50 anos, e cinco, com mais de 51 anos.

Em relação ao tempo de trabalho, evidenciou-se que 16 participantes apresentavam experiência há mais de dois anos na unidade de análise investigada e sete participantes declararam experiência menor que um ano. Em relação ao tempo de formação, apenas três relataram período menor que dois anos. Especificamente na APS, como enfoque deste manuscrito, sete profissionais tinham experiência menor que dois anos na unidade investigada.

A respeito dos cuidadores familiares, entre os seis participantes, apenas um entrevistado representava o sexo masculino. Quanto à idade, dois participantes apresentavam entre 20 e 40 anos, três, de 40 a 60 anos, e apenas um, acima dessa faixa etária. Em relação ao grau de parentesco, quatro eram filhos, uma era esposa e outra se identificou como neta.

Esta pesquisa qualitativa obteve um volume significativo de resultados, que foram analisados a partir de 777 citações. As categorias originadas foram exploradas posteriormente, abrangendo diferentes nuances da APS. Neste artigo, apresenta-se o aprofundamento da categoria “Atuação da Atenção Primária à Saúde na transição do cuidado”, que foi relacionada a 298 citações, devido à sua relevância destacada no contexto da transição do cuidado e para responder ao objetivo proposto.

O envolvimento da APS no contexto da continuidade do cuidado a pessoas com AVC após a alta hospitalar foi abordado com todos os participantes do estudo, e os resultados retratam alguns desafios nesse ponto de atenção, como o afastamento dos preceitos básicos do serviço, a busca da promoção da saúde e a prevenção de agravos, devido a um cenário com enfoque em questões curativistas e que demandam atendimento imediato após o adoecimento. A alta procura na demanda espontânea das unidades gera ansiedade nos profissionais que, muitas vezes, não conseguem prestar o cuidado longitudinal, conforme relatado na fala abaixo.

*E muitos não entendem ainda o atual conceito de Atenção Primária à Saúde. Não entendem que é prevenção. A gente só trabalha o problema. E eu acho que a gente tá perdendo essa essência de Atenção Primária à Saúde. Muitos pacientes caracterizam que aqui é um PA* [Pronto Atendimento], *então acho que a gente tá perdendo um pouco isso.* [...] *eu estou cansada de apagar incêndio, eu quero conhecer a família que eu atendo.* (APS7)

Profissionais de outros serviços também compreendem a necessária continuidade de cuidados na APS como o único caminho para impactar a redução da incidência do AVC e a prevenção secundária suficiente, evitando a recorrência da doença.

*Se tu aumenta leito no hospital, quer dizer que a tua Atenção Básica tá ruim. Só que, para tua Atenção Primária melhorar, quando você devolve o paciente, o que se espera é o controle dos fatores de risco, é fazer o monitoramento desse caso para evitar que ele tenha uma recorrência, senão o hospital vai ter que começar a aumentar leito.* (SA2)

De acordo com a Linha de Cuidado municipal, o profissional médico ou enfermeiro da unidade de saúde deve realizar o primeiro atendimento. Esse cuidado em saúde deve promover a continuidade do acompanhamento, incluindo tratamento adequado de acordo com a etiologia do evento, identificando e atuando nos fatores de risco que são modificáveis e possibilitando a inserção da pessoa em programas existentes, como grupos para pessoas com hipertensão e diabetes, estímulo à atividade física e controle do tabagismo. Nas falas, pôde-se observar um olhar integral dos participantes, que perpassa a medicalização.

*Eu tento mostrar para o paciente que vai muito além da medicação* [...] *se a gente informa o paciente, é muito mais fácil de ele seguir o que a gente tá falando, né? Se a gente pede exame que ele não sabe nem pra que que é, bem dificilmente esse paciente retorna.* (APS7)

Os profissionais percebem a transição do cuidado como uma assistência continuada necessária que, mesmo após a desospitalização, mantém-se na rede com o olhar atento da APS. A fala abaixo retrata o que os profissionais elencam como prioridade após o AVC e a alta hospitalar.

*O principal é ficar cuidando das comorbidades para tentar evitar novos eventos. Muitos deles não têm o conhecimento de que o bom controle das comorbidades é realmente diminuir o risco dos eventos, então a gente realmente tenta orientar e educar bastante a importância do controle da pressão, diabetes, tabagismo* [...]. (APS2)

Cita-se também a importância da inserção da família nas práticas em saúde e planejamento dos cuidados, compreendendo que possuem importante papel nesse contexto. Além disso, a APS retrata a possibilidade de maior vínculo com as pessoas e o compartilhamento das decisões.

*A gente precisa também prevenir fatores de risco, porque, se não, é um forte candidato a ter novas complicações. Então, para mim, é um cuidado integral, é uma linha de cuidado mesmo que a gente deve seguir e que aí a gente encontra o profissional tanto do nível terciário, secundário e primário. A gente tem que trazer de forma não vertical, mas mais horizontal, para poder dar uma assistência de qualidade adequada não só para ele, mas para a família também.* (APS8)

Embora não esteja sinalizado nos fluxos instituídos, a atuação da equipe multiprofissional foi trazida pelos participantes como importante ponto de apoio na transição do cuidado, incluindo estímulo às mudanças no estilo de vida e reabilitação adaptada à cada situação. A fala de uma cuidadora salienta a importância da atuação multidisciplinar para hábitos mais saudáveis.

*A glicose da mãe estava bem descompensada. A gente já tinha tentado de outras formas mudar a alimentação, mas não radicalmente. Agora, com a nutricionista, cortou tudo: açúcar, carboidrato* [...] *diabetes é uma doença silenciosa, né, é uma doença que não existe um tratamento milagroso. Tu precisa mudar o teu estilo de vida.* (CF4)

*O nosso fisioterapeuta do NASF* [Núcleo de Apoio à Saúde da Família] *faz a visita domiciliar para o paciente, mas não é com aquela regularidade, até porque é só um fisio e a demanda é enorme. Ele tenta mostrar para a família o que eles podem usar dentro das condições deles. Para a melhora do paciente, a questão de colchão, posicionamento, troca de decúbito* [...]. (APS7)

Os entraves organizacionais, como a escassez de profissionais, são citados por alguns participantes. Todavia, observou-se que as equipes realizam novos arranjos, de modo a conseguir oferecer o serviço adequado à pessoa desospitalizada, como na fala relatada abaixo.

*A gente tinha um terapeuta ocupacional* [TO] *e dois psicólogos. Agora, a gente tem um psicólogo só. Mas a gente tem um engajamento muito grande com o paciente. É nossa responsabilidade. Às vezes, eu não tenho um TO, mas se eu estou precisando, a coordenadora joga no grupo das coordenações e o pessoal ajuda, sabe? Da mesma forma que a gente tem gineco e pediatra, a gente ajuda os outros também. A troca entre equipes e a responsabilidade que se tem com o paciente, eu acho muito legal.* (APS10)

Em relação às estratégias realizadas pela APS, cita-se a promoção da saúde, envolvendo principalmente ações coletivas, como os grupos realizados. Embora a pandemia de COVID-19 tenha impossibilitado os encontros, os profissionais destacaram a importância dessas ações para interferir positivamente nos fatores de risco do AVC.

O papel do profissional enfermeiro é realçado nas falas dos participantes que atuam na APS, resgatando o olhar integral e atento da categoria no gerenciamento do cuidado das pessoas com AVC.

*Por mais que o ACS, o médico, todo mundo visite, o enfermeiro é que traça um plano para esse paciente. A gente é quem procura ver realmente quais são as necessidades e enxerga a família como um todo* [...] *todo o cuidado de prescrever os materiais especiais do curativo, avaliar, fazer um acompanhamento, levar a discussão para a equipe multi, envolver toda a equipe, estar um pouco mais à frente dessa família. Eu vejo que o enfermeiro que coordena todo esse cuidado. A gente é meio condutor desse processo. Percebo que a enfermagem também acaba fazendo como se fosse um “Projeto Terapêutico Singular” ali, para aquela família, para aquele usuário, nesse momento. Porque ela já deixou o agente comunitário orientado a passar mensalmente para ver como que está; se achar que tem uma necessidade do técnico ir, a gente também já dá esse desfecho. A gente acaba fazendo alguns controles, que são do próprio perfil da enfermagem. A gente tem essa visão um pouco mais ampliada do cuidado.* (APS8)

*Normalmente, a enfermeira acaba recebendo essa informação primeiro* [contrarreferência], *até porque a gente acaba atuando mais nos agendados. Ela acaba recebendo a contrarreferência e já agenda uma visita domiciliar para poder fazer avaliação do paciente. Às vezes, até uma consulta mesmo na unidade se o paciente tiver condições de vir. Depois, ela acaba me passando isso, na própria equipe, pra gente ou fazer uma visita também ou para marcar uma consulta e acompanhar esse paciente.* (APS6)

*Avaliar o paciente e quais foram as necessidades, quais foram as limitações, sequelas, saber como o paciente está e saber quais os cuidados que ele precisa. A partir desses cuidados que a gente avalia para fazer o plano de cuidado dele, preparar e organizar junto à família.* (APS5)

Além da importância do papel da enfermagem nos serviços de saúde, no trabalho das Unidades Básicas de Saúde, soma-se o vínculo com as pessoas e suas famílias, permitindo que a equipe se torne uma referência à comunidade quando buscam orientações ou qualquer outro tipo de assistência. Todavia, algumas barreiras são encontradas pelos familiares quando buscam por auxílio e informações, conforme descrito abaixo.

*A equipe do postinho anda muito desfalcada. É uma das maiores dificuldades que a gente tem hoje em dia é quando você se acostuma com uma equipe, ela vai pra outro lugar, daí você tem que se acostumar tudo de novo. Com aquela enfermeira, já estava acostumado, era a pessoa que mais ajudava a gente antes do AVC dele e depois muitas coisas eu ainda conversei com ela, aí ela foi para o outro posto* [...] *esse mês, estava com medo de não conseguir pegar os materiais. Daqui pra frente, já não sei qual vai ser a enfermeira que vai cuidar dele* [familiar que teve o AVC]*, então vou ter que começar tudo de novo. Lógico que deve ter no sistema muitas informações. A gente fica um pouco perdido. Que nem o médico, se você vai duas vezes nele, na terceira vez que você chega, ele já te conhece um pouco, né? Aí se muda, começa tudo de novo. Dificulta um pouco pra gente.* (CF5)

Com a construção da Linha de Cuidados do AVC no município estudado, buscou-se aprimorar a continuidade do cuidado e assistência integral à pessoa acometida, incluindo no seu retorno para casa. Os primeiros dias após a alta hospitalar representam o momento mais estressante para as famílias, surgindo muitas dúvidas e inseguranças com o cuidado a ser realizado. Profissionais que atuam no ambiente hospitalar salientam a necessária brevidade da continuidade de cuidados, prestada pela APS, conforme observado nas reuniões da equipe. Durante a coleta em um dos serviços de apoio, uma profissional relata que, durante as ligações telefônicas de acompanhamento, observa que algumas pessoas não conseguem acesso aos serviços para consulta em tempo recomendado.

*A gente precisa pensar em alguma estratégia, de ter alguém que seja a referência para esse paciente, um profissional que vai ajudar ele nessa jornada, porque ele vai chegar em casa cheio de dúvidas e ele não vai poder ligar na Unidade de AVC e falar com a enfermeira pra ter o suporte que ele precisa. Ele vai na Atenção Básica, vai ser acolhido, mas vai entrar numa agenda que talvez não responda à dúvida imediata dele, e a consulta vai ficar para daqui a 15 ou 20 dias.* (SA2)

Além da garantia de acesso nos cuidados primários à saúde, urge a importância de um atendimento integral, mas que também atenda às especificidades de ações após o acontecimento do AVC e suas implicações. Envolve o engajamento do usuário e seus familiares no cuidado, ultrapassando o modelo biomédico e centrado apenas na medicalização e solicitação de exames, o que pode demandar um esforço do profissional que o auxilia, para sensibilizá-los na continuidade do cuidado, prevenção secundária e demais hábitos saudáveis necessários.

*E eu não coloco só a conta para a população. Às vezes, nós mesmos. A gente também faz errado. Às vezes, é muito mais fácil você entregar uma folha com hemograma completo do que ter que explicar pra ele que ele precisa comer melhor.* (U-AVC7)

[...] *a orientação do nutricionista na alta para reeducação alimentar é uma orientação muito básica, muito geral, não é individualizada. Então, muitas vezes, não surte efeito para mudança de hábitos, né? Precisa ter alguma coisa, um acompanhamento mais próximo, um programa, grupo ou programa individual com metas.* (U-AVC1)

No contexto da transição do cuidado, um ponto citado pelos participantes foi a respeito do papel do ACS nesse processo de transição do cuidado. As falas abaixo retratam esse profissional como uma figura importante nas equipes de Saúde da Família, pois favorecem o vínculo, possuem conhecimento do território e reconhecem os agravos, levando aos demais membros da unidade de saúde informações relevantes que podem ser transmitidas previamente à notificação de contrarreferência.

*A gente costuma usar muito as Agentes Comunitárias de Saúde, que fazem também esse acompanhamento dos usuários. Elas também fazem as visitas e acabam descobrindo* [pessoas após o AVC]. *Às vezes, nem é um paciente que teve AVC aqui, mas é um paciente que fez um AVC em outro município e acabou vindo por conta dos familiares, e a gente acaba descobrindo isso através dos ACS.* (APS6)

*Geralmente, a gente acaba sabendo por meio das ACS que o paciente esteve internado por causa de AVC, aí já pede para um familiar acionar a unidade para marcar consulta de retorno após a internação.* (APS11)

*O postinho conhece muito a gente. Elas realmente são muito legais para conseguir as coisas, sabe. Então, a gente bate muito papo, a gente conversa de outros lugares, de academia que a gente já se conhece. Elas são muito atentas aqui em casa. Sabem que o meu filho é autista, que o pai teve AVC, e perguntam por eles. Posso dizer que elas correram atrás para conseguir a fisio, para conseguir as coisas. Ela é muito, muito atenciosa. Elas estão passando direto, então, quando tem alguma dúvida, eu já pergunto.* (CF3)

Os participantes também relataram que, apesar da relevância do ACS para a saúde das famílias, a escassez de profissionais, a falta de cobertura das microáreas e outras tarefas realizadas pela categoria afastam a possibilidade da atuação ideal desse profissional no território. As falas quanto às dificuldades enfrentadas com a pandemia de COVID-19 são corroboradas entre profissionais e cuidadores.

*Temos as Agentes Comunitárias que passam nas casas, e, em função da pandemia, essa rotina tá toda alterada*. (APS5)

*Com a pandemia, muita coisa mudou. Eles não estavam na rua, alguma coisa a gente não sabia, e aí o próprio familiar vinha até aqui pra explicar. Até quando precisava de alguns encaminhamentos, eles acabam vindo* [...]. *O ACS, por falta de pessoal, começou a ficar dentro do posto sem poder fazer suas visitas. Aqui eles não tinham mais o que trazer para nós, o que estava acontecendo na rua. A gente tinha algumas coisas que o familiar vinha depois trazer, mas os ACS não estavam sempre ali presentes.* (APS3)

*Com a pandemia, não teve mais visitas da ACS, e essas equipes agora estão muito desfalcadas.* (CF5)

Ainda que haja uma Linha de Cuidado estabelecida e a criação de um fluxo de contrarreferência entre hospital e serviços de APS, salienta-se o reconhecimento do AVC como um agravo tão importante quanto outras demandas de cuidado, comparado à saúde das crianças, pré-natal, tuberculose, entre outros.

*A gente não tem essa cultura, no nosso país, de valorizar a Atenção Primária. A gente percebe como que os índices melhoram, diminui mortalidade, como você controla bem hipertensão, controla bem o diabetes, sabendo que você vai resolver na Atenção Primária 80% dos motivos de consulta do teu paciente, você fortalecer a figura do médico de família, do enfermeiro, da equipe que trabalha ali na estratégia.* (U-AVC7)

Ao questionar os profissionais a respeito da Linha de Cuidado, observou-se fragilidade no conhecimento e leitura do material, justificado por alguns participantes devido à alta demanda de atendimentos e sobrecarga de trabalho.

*Não consegui ler. É tanta coisa que chega, então ler tudo aquilo e mais 25 ou 30 pacientes por dia fica complicado, sabe?* (APS4)

## DISCUSSÃO

A APS é o serviço de referência para os usuários após a alta hospitalar, pois a equipe situa-se em um ponto da rede que proporciona maior vínculo e abordagens às pessoas e seus familiares, além de possibilitar a participação do cuidado e compartilhamento das decisões. É importante que as famílias identifiquem onde poderão buscar apoio e quem são os profissionais de sua referência para auxílio nos cuidados após a alta, sinalizando quais são os serviços disponíveis na rede para o processo de reabilitação. As ações também incluem o apoio e o direcionamento de demandas que ultrapassam questões físicas, muitas vezes esquecidas pelos profissionais^(2,5)^.

Outros elementos são relevantes na percepção de quem sobreviveu ao AVC, como poder voltar a estudar, ter autonomia para as atividades básicas da vida diária, manter a socialização com amigos e familiares e compreensão acerca da doença. A transição do cuidado adequada para a APS deve promover informações relevantes a respeito das condições de saúde e riscos de um novo evento, orientar o uso correto dos medicamentos e estimular hábitos saudáveis, como atividade física, cessação do tabagismo e cuidados com a alimentação^(4)^, fato que foi observado no caso estudado.

Algumas iniciativas foram elencadas pelos participantes envolvendo estratégias para hábitos de vida mais saudáveis, como orientações acerca da alimentação, controle da pressão arterial e tabagismo. Estudo brasileiro avaliou os efeitos do Programa Academia da Saúde nos gastos com internações por AVC, e, entre seus achados, observou-se redução dos custos em municípios que aderiram ao programa, o que reforça a importância de políticas de promoção da saúde voltadas para mudanças no estilo de vida^(13)^.

Ações que não se restrinjam apenas às pessoas com maior risco cardiovascular são sustentadas. Intervenções não farmacológicas, como o incentivo às mudanças do estilo de vida e políticas públicas saudáveis, são reforçadas pela literatura como estratégias benéficas. Os serviços de saúde também podem oportunizar a troca de experiências entre cuidadores familiares. Essa estratégia pode auxiliar que encontrem, coletivamente, ferramentas para enfrentamento ao longo de todo o cuidado e reabilitação^(14)^, incluindo desenvolvimento de habilidades, possibilitando voz às famílias diante dos desafios deixados pela doença. É necessária uma mudança de olhar dos que atuam na assistência à saúde, compreendendo que todas as pessoas com qualquer risco de AVC e doença cardiovascular devem ser alvo para promoção da saúde, não apenas grupos considerados com maior risco^(15)^.

Visando ao aumento da resolutividade dos serviços primários, as equipes multiprofissionais ampliam a atenção à saúde das pessoas, incorporando em sua essência a integralidade do cuidado. Além disso, o ACS é descrito neste estudo como uma ponte entre a comunidade e os serviços prestados pelas unidades de saúde. Sabidamente conhecido como uma categoria que atua na prevenção de doenças e promoção da saúde, também sofreu interferências com a atualização da Política Nacional de Atenção Básica. A descaracterização do papel desse profissional ocorre com a vinda de procedimentos em seu escopo de atuação, como aferição de sinais vitais e técnicas de curativo simples^(16)^, podendo fragilizar outras ações no território.

Como parte do escopo das Unidades Básicas de Saúde, os programas de visita domiciliar configuram-se prática singular da Atenção Básica, principalmente quando envolve a equipe multiprofissional, que auxilia na transição adequada do cuidado por sua possibilidade de ampliar o olhar à integralidade do cuidado aos aspectos que ultrapassam a doença^(17-18)^.

Ensaio clínico randomizado^(19)^ percebeu que o grupo de pessoas e familiares que receberam visitas de profissionais enfermeiros no domicílio após a alta apresentou maior acesso no serviço ambulatorial hospitalar, o que pode ser justificado pelo maior vínculo com o ponto da rede e efetividade nas orientações. As visitas contemplavam educação em saúde, orientações sobre o sistema e demonstrações reais acerca do cuidado para o desenvolvimento de habilidades. Além disso, a família era informada sobre serviços onde poderia conseguir auxílio em materiais e insumos para o cuidado, sendo a principal preocupação das famílias nos primeiros dias após a alta.

Na visita domiciliar, também é possível identificar os DSS, incluindo a avaliação da alimentação adequada, situação socioeconômica, estrutura física e apoio familiar^(20)^. Além disso, realizar orientações adaptadas às realidades familiares fortalece o engajamento para o cuidado e o compartilhamento das decisões^(21)^. Apesar de as visitas fazerem parte do escopo das equipes, durante a coleta, não se observou como as equipes se programam para a continuidade dos acompanhamentos.

A respeito das informações em saúde, recomenda-se que a comunicação seja de forma clara, incluindo quais serviços estão à disposição das pessoas, compreendendo o contexto que vivem. Serviços de tecnologia em saúde são bem avaliados, como a contrarreferência utilizada entre serviço hospitalar e de APS, pois podem possibilitar o acompanhamento rotineiro dos novos casos de AVC e organização das equipes para atenção a essas pessoas. Além disso, a telessaúde pode ser vantajosa na colaboração entre profissionais^(22)^.

A permanência das taxas de mortalidade em algumas regiões do mundo sugere políticas públicas mais efetivas, principalmente com enfoque na prevenção primária e secundária. Por outro lado, entende-se que os países com redução nas taxas brutas de mortalidade ao longo dos anos tiveram desenvolvimento das estratégias de manejo e tratamento do AVC, como a criação de unidades de AVC e identificação dos casos mais leves^(23)^. Isso reforça a atuação das equipes hospitalares, como observado no estudo, que possuem olhar direcionado à alta e ao preparo do cuidador desde o momento da hospitalização.

Linhas de Cuidado bem estabelecidas e fluxogramas dos serviços são essenciais para a garantia da transição adequada, embora não sejam suficientes. O planejamento do cuidado deve se fazer cumprir em todos os pontos que prestam assistência ao indivíduo com AVC, e a qualidade prestada na fase aguda do evento deve ser continuada nos demais pontos da Rede de Atenção à Saúde.

Embora tenha sido observado o entendimento a respeito do papel dos serviços de APS, os profissionais se deparam com dificuldades nas suas rotinas. Esse fator também compromete a experiência e o acesso das pessoas e familiares que vivem com o AVC. Os entraves identificados corroboram a literatura, como a alta demanda nas portas das unidades para atendimentos pontuais, a superlotação, as dificuldades no acesso à reabilitação, os limites geográficos, as questões estruturais e de dimensionamento de equipe, o impacto da pandemia e a mudança nas políticas de saúde. É um contexto que desafia a articulação da equipe multiprofissional para a continuidade adequada do cuidado na complexidade do AVC, especialmente por requerer abordagens distintas^(3)^.

A saúde é determinada e condicionada por diversos fatores que ultrapassam mudanças comportamentais. Os caminhos para os avanços em saúde fundamentam-se na promoção da saúde, medidas preventivas e melhores condições de vida. Requer olhar positivo para a saúde, políticas públicas saudáveis, reorganização da rede de saúde, envolvimento da comunidade na participação das decisões, ambientes saudáveis e desenvolvimento das habilidades pessoais^(24-25)^.

### Limitações do estudo

O estudo foi desenvolvido em uma cidade reconhecida pelo cuidado à pessoa com AVC, com habilitação na Portaria nº 665 de 2012, como Centro de Atendimento de Urgência Tipo III^(26)^. Além disso, conta com programa de residência multiprofissional e instiga constantemente o aprimoramento do cuidado às pessoas com AVC, o que contribui fortemente para a transição do cuidado. Elenca-se como limite do estudo a generalização dos dados para outras realidades. Além disso, observaram-se as implicações da pandemia de COVID-19 nas rotinas das equipes, interferindo nas atividades profissionais e, em suma, determinando achados que poderiam ser distintos em outras condições sanitárias.

### Contribuições para as áreas da enfermagem, saúde e política pública

Os resultados da pesquisa ampliam as perspectivas de atuação da enfermagem na promoção da saúde em contexto hospitalar, valorizando a atuação do enfermeiro de transição. Tem relevância para a formação profissional e o trabalho multiprofissional no SUS, com vistas à Linha de Cuidado em AVC e o cuidado na Rede de Atenção à Saúde. A pesquisa fornece reforços de compreensão da gestão em saúde quanto à avaliação dos resultados de fluxos estabelecidos, instigando a articulação entre os pontos da Rede de Atenção à Saúde, com criação de políticas públicas para a redução da mortalidade pelo AVC e para a coordenação do cuidado após a alta na APS.

Os primeiros dias após a alta são o momento mais delicado para as famílias, urgindo a atuação em tempo oportuno para identificar as necessidades da família. Reflete-se sobre a possibilidade de programas de cuidados de transição que possibilitem a continuidade dos cuidados sem que haja lacunas entre os pontos da rede. Além da criação de Linhas de Cuidado, também se faz necessária a educação permanente para capacitação das equipes para assistência, possibilitando a transição de cuidado.

## CONSIDERAÇÕES FINAIS

A atuação da APS no cuidado às pessoas após o AVC contempla oportunidade de acesso aos serviços, prevenção secundária e estratégias promotoras de saúde que visem ao empoderamento e à autonomia das pessoas, com a melhoria dos seus estilos de vida, a vigilância realizada pelo ACS e a atuação multiprofissional para garantia da integralidade do cuidado. Ademais, enfatiza-se o papel protagonista da família e do profissional enfermeiro como gestor do cuidado.

Apresentam-se como desafios a alta carga de trabalho nas unidades, os DSS e a pandemia de COVID-19. Recomendam-se novos estudos em outras realidades, contemplando a identificação de arranjos distintos na Linha de Cuidado à pessoa com AVC que possam levantar estratégias relevantes para a garantia da transição do cuidado.
