# Supplementary material for: ERRATUM: Primary Health Care in transitional care of people with stroke
Source: Rev Bras Enferm. 2026 May 27;79:e2026e0420230468. doi: 10.1590/0034-7167.202679e0420230468 (PMC13480450; doi:10.1590/0034-7167.202679e0420230468)
Supplement: Related texts

# Correction: ERRATA: Atenção Primária à Saúde na transição do cuidado de pessoas com Acidente Vascular Cerebral

**DOI:** 10.1590/0034-7167.202679e0420230468pt

No artigo “Atenção Primária à Saúde na transição do cuidado de pessoas com Acidente Vascular Cerebral”, com número DOI: https://doi.org/10.1590/0034-7167-2024-0468pt, publicado no periódico Revista Brasileira de Enfermagem, 2024;77(3):e20230468:

Onde se lê:

https://doi.org/10.1590/0034-7167-2024-0468pt

Leia-se:

https://doi.org/10.1590/0034-7167-2023-0468pt

Profa. Dra. Dulce Barbosa

*Editora-Chefe*
